# The Prevalence of Dietary Supplement Use Among Elementary, Junior High, and High School Students: A Nationwide Survey in Japan

**DOI:** 10.3390/nu10091176

**Published:** 2018-08-28

**Authors:** Etsuko Kobayashi, Chiharu Nishijima, Yoko Sato, Keizo Umegaki, Tsuyoshi Chiba

**Affiliations:** 1 Department of Food Function and Labeling, National Institute of Health and Nutrition, National Institutes of Biomedical Innovation, Health and Nutrition, 1-23-1 Toyama, Shinjuku-ku, Tokyo 162-8636, Japan; e-kbyshi@nibiohn.go.jp (E.K.); c-nishijima@nibiohn.go.jp (C.N.); satoyoko@nibiohn.go.jp (Y.S.); 2 Laboratory of Medical Chemistry, Graduate School of Nutrition Sciences, Kagawa Nutrition University, 3-9-21 Chiyoda, Sakado, Saitama 350-0288, Japan; 3 Department of Food Safety and Management, Showa Women’s University; 1-7-57 Taishido, Setagaya-ku, Tokyo 154-8533, Japan; k-umegaki@swu.ac.jp

**Keywords:** dietary supplement, children, mothers, adverse event, internet survey

## Abstract

The prevalence of dietary supplement use, such as vitamins, minerals, or fish oil, has increased among children in Japan; however, whether children are using dietary supplements appropriately remains unclear. This study aimed to determine dietary supplement use among children. In August 2017, a nationwide internet preliminary survey of 265,629 mothers aged from 25 to 59 years old was undertaken. Of these, 19,041 mothers of children attending either elementary school, junior high school, or high school were selected. Among them, 16.4% were currently providing their children with dietary supplements and 5.2% had previously given dietary supplements to their children. The prevalence of dietary supplement use was higher in boys than in girls, and the prevalence increased according to their grade. A total of 2439 participants were eligible to undertake a targeted survey on dietary supplement use. Dietary supplements were being taken to maintain health, supplement nutrients, and enhance growth in both boys and girls, and many children (37.5%) were provided with vitamin and mineral supplements. Mothers mainly obtained information concerning dietary supplements via the internet, and supplements were purchased in drug stores or via the internet. The prevalence of dietary supplement use in mothers was 65.4% and may be associated with the prevalence rates in children. Some mothers reported adverse events (3.6%) in their children, such as stomachache, diarrhea, nausea and vomiting, and constipation. The cause-and-effect relationships for adverse events were not clear, but some children were given products for adults. Children are more influenced by dietary supplements compared to adults. To prevent adverse events due to inappropriate use, parental education concerning dietary supplements is essential.

## 1. Introduction

The prevalence of dietary supplement use for added nutrients has increased in Japan [1,2]. Several prevalence studies concerning dietary supplements have been conducted not only in Japan [3], but elsewhere [4,5,6]. The characteristics of dietary supplement users are similar among countries. The factors associated to dietary supplement use are sex, age, education level, and socioeconomic status [4,7,8]. Many studies have focused on elderly populations [7,9,10] or patients [11,12], because these populations are inclined to use dietary supplements for their health and may use dietary supplements instead of medicines to treat diseases. Previously, our group reported that the prevalence of dietary supplement use among preschool children aged from 0 to 6 years was between 8.0% [13] and 15.5% [14] in Japan. It has been reported that parents provide dietary supplements to preschool children mainly because they consider their children do not derive sufficient nutrients from meals. However, they provide their children not only with vitamin/mineral supplements, but also herbs or other ingredients [14]. Inappropriate usage of dietary supplements in children is concerning because dietary supplement use more readily affects children than adults.

Previously, we conducted a nationwide survey to determine the prevalence of dietary supplement use among college students in Japan. The prevalence of dietary supplement use among college students was 16.8%, and prevalence increased from 1st grade (13.8%) to 4th grade (18.7%), in accordance with their school grade [15]. Unlike preschool children, most students had purchased the dietary supplements themselves for intended purposes such as building muscle, weight loss, as a beauty product, or for additional nutrient supplementation. Some students had experienced adverse events associated with dietary supplement use, despite previous college education concerning nutrients and dietary supplement use. Recognition of dietary supplements might be acquired before this period.

At this time, there are no reports about the prevalence of dietary supplements from elementary school to high school students in Japan. Therefore, we conducted a nationwide survey on mothers whose children were elementary, junior high, or high school students to clarify the prevalence of dietary supplement use in these generations.

## 2. Materials and Methods

### 2.1. Internet Survey and Participants

An internet-based survey was conducted, as described previously by Macromill, Inc. (Tokyo, Japan), between 4 August and 28 August 2017 [15]. A total of 265,629 mothers aged from 25 to 59 years old who had school-aged children were invited to participate in this survey and 19,041 mothers responded to the preliminary survey. Of these, 2439 mothers whose children were current- or ex-users of dietary supplements completed the targeted survey. Mothers who had multiple school-aged children were asked to answer only in response to their oldest child. Children were classified according to sex, as well as school grade: elementary school (ES) (1st-3rd grade), and ES (4th-6th grade), junior high school (JHS), and high school (HS). This study was conducted with the approval of the Research Ethics Committee of the National Institutes of Biomedical Innovation, Health and Nutrition (No. 119, approved on 15 May 2017) and in accordance with the Declaration of Helsinki.

### 2.2. Questionnaires for the Preliminary and Targeted Surveys

Throughout the whole survey process, a “dietary supplement” was defined as a supplement that respondents considered had beneficial effects on human health (e.g. vitamins, minerals, fish oil, amino acids) apart from conventional foods (e.g. vegetables, fruits, milk). Demographic information (resident area, household income, sex, and school grade of their child) and information on the children’s dietary supplement use (“currently using”, “previously used”, or “never used”) were obtained in the preliminary survey.

In the targeted survey, the questionnaire included the same questions as our previous study. Information was gathered concerning the number of supplement products used at a time, the information sources in regard to dietary supplements, how products were obtained, the purpose of use, the types of dietary supplements, adverse event experiences, and responses to adverse events. Questions on new topics were also added, such as the mothers’ dietary supplement use (“currently using”, “previously used”, or “never used”), awareness of dietary supplement effectiveness, and the mothers’ perceptions in regard to dietary supplements. The questionnaire consisted of 14 topics assessing the safety and effectiveness of dietary supplement use, and used a 5-point Likert scale that ranged from “strongly agree” to “strongly disagree”. All questions were designed so that “strongly disagree” responses were deemed the most preferable answers. For example, supplement providers generally encourage consumers dietary supplement use with the exploitation of “Dietary supplements are safe because they are just foods” or “Dietary supplements made from natural ingredients or herbs are safe”, even though they do not manage the safety of their products. In this regard, “strongly disagree” shows the preferable perception. In the other case of “Food additives should be avoided”, the amounts of all food additives added in foods are strictly regulated to be below Acceptable Daily Intake; thus, we do not need to overly avoid them. However, most of consumers have a misperception that food additives induce cancer regardless of the kinds and the amount of food additives. In the same manner, the most preferable answers are also set to be “strongly disagree” for the rest of the topics, considering our situation in Japan.

### 2.3. Statistical Analysis

A chi-squared test was used to assess differences among groups (the sex of children or school grade levels). All statistical analyses were performed using Stata/IC 15 (Light Stone, Tokyo, Japan) and a *p*-value of <0.05 was considered statistically significant.

## 3. Results

### 3.1. Characteristics

The characteristics of the children of mothers who completed the preliminary survey (*n* = 19,041, boys: 9791; girls: 9250) and the targeted survey (*n* = 2439, boys: 1236; girls: 1203) are shown in Table 1. The participants of each grade (ES (1st-3rd), ES (4th-6th), JHS, and HS), distributions of residential area, and household income in both the preliminary and targeted surveys are also shown in the table. House income was slightly different from general income in Japan. The ratios of 4–6 million yen (25.7%) and 6–8 million yen (16.6%) were higher than the general income (19.5% and 13.6%, respectively).

### 3.2. The Prevalence of Dietary Supplement Use

The prevalence of dietary supplement use in children whose mothers answered the preliminary survey is shown in Table 2. Among 19,041 children, 3127 (16.4%) were currently using dietary supplements, 998 (5.2%) had taken them in the past, and 14,916 (78.3%) had never taken them. There was a significant gender difference in the prevalence of dietary supplement use, with the ratio of current users being higher in boys than in girls across all school grades. In addition, the prevalence rate increased as their grades advanced for both sexes (current users, from 12.4% in ES (1st-3rd) to 21.3% in HS; previous users, from 3.0% in ES (1st-3rd) to 7.9% in HS). The prevalence of current users also increased according to household income (data not shown; *p* for trend <0.01).

### 3.3. The Concomitant Use of Dietary Supplements and Frequency of Dietary Supplement Use

We asked mothers about the number of dietary supplements their children took concomitantly. Although most children (72.8%) were taking 1 product, more than a quarter of the children were using 2 or more products concomitantly. In addition, 19 (0.8%) mothers gave more than five products to their children. We also asked questions in regard to the frequency of dietary supplement use. Half (50.2%) the mothers gave dietary supplements almost every day, followed by three to five days a week (24.7%), two or three days a week (12.7%), and several times a month (4.8%). On the other hand, 6.1% of mothers answered that they gave dietary supplements only when they felt it necessary.

### 3.4. Information Sources and How Dietary Supplements Were Obtained

Information sources concerning dietary supplements and how they were obtained are shown in Table 3 and Table 4, respectively. In mothers of both boys and girls, the most popular way to obtain information about dietary supplements was via the internet (40.4%); however, more boys’ mothers obtained information from friends or acquaintances (17.5% in boys vs. 14.2% in girls, *p* = 0.03) and from coaches of clubs (3.7% in boys vs. 0.7% in girls, *p* < 0.01). More girls’ mothers obtained information from pharmacists or drug store clerks (8.7% in boys vs. 11.5% in girls, *p* = 0.03). In terms of information sources, the internet was used most often to purchase dietary supplements in boys’ mothers (42.3%) and the pharmacy or drugstore for girls’ mothers (46.0%). More boy’s mothers also obtained information from sports stores (3.2% in boys vs. 0.8% in girls, *p* < 0.01) and sports club (0.9% in boys vs. 0.2% in girls, *p* = 0.04).

### 3.5. The Purposes of Dietary Supplement Use and the Types of Supplements Used

The purposes of dietary supplement use are shown in Table 5. These were expected to differ according to school grade, so we undertook analyses through further dividing the children into school grades. Maintenance of health, supplementation of nutrients, and to enhance growth were the three most frequent responses concerning the purposes of dietary supplement use in both boys and girls and in all grades, except for the response to ‘Enhance growth’ in HS girls (8.1%). The responses to the topic ‘Enhance stamina’ tended to show an increase in use according to advancing grades in both boys and girls (from 12.0% in ES (1st-3rd) to 32.7% in HS for boys, from and 8.5% to 12.3% for girls), as well as the case with responses to ‘Enhance athletic performance’ in boys (2.6% to 19.7%), and with responses to ‘Weight loss’ in girls (1.8% to 4.5%).

### 3.6. Types of Dietary Supplements Used

The types of dietary supplements used by children are shown in Table 6. Users of any types of vitamins and minerals accounted for 31.1% to 53.7% depending on their sex and grades. Individual vitamins such as vitamin C and B vitamins, and minerals such as calcium and iron, were used more than premixed multi-vitamins and multi-minerals. The use of individual minerals in girls increased with advancing grades (14.9% in ES (1st-3rd) to 26.5% in HS, *p* < 0.01), and the mineral mostly used in the HS girls was iron. Growth-promoting supplements were consistently used from ES (1st-3rd) until JHS, and rates ranged from 15.9% to 23.9% in boys to between 11.3% and 13.8% in girls, but the use decreased in HS (9.1% in boys and 1.3% in girls). Instead, protein/amino acid supplement use gradually increased according to grades and reached 33.3% in HS boys. More younger children tended to use probiotics and aojiru. Cod liver oil was popular specifically in ES (1st-3rd) boys (12.9%) and girls (12.8%) compared to those in other grades (ranged from 1.0% to 3.9%). The prevalence of weight loss supplement use in girls was only 0.0% to 2.3%. In addition to these major products, and other various kind of dietary supplements such as glucosamine, chondroitin, garlic, propolis, or some herbal products, were used by a few children. Therefore, these products were included in “Others” in Table 6.

### 3.7. Awareness of Effectiveness and Adverse Events

Regarding the effectiveness of dietary supplements, 28.1% of mothers answered that the dietary supplements they gave their children were effective and only 3.3% answered that they were ineffective. In contrast, 68.6% of mothers answered “unsure” as to whether the dietary supplements their children used were effective or not.

The prevalence and symptoms of adverse events due to dietary supplement use are shown in Table 7. In total, 3.6% (88/2439) of children were reported to have experienced adverse events in this survey. There was no difference between boys (3.7%) and girls (3.5%), but the prevalence was higher in younger children (5.4% in ES (1st-3rd) vs. 2.1% in HS). Among them, stomachache (23.9%) and diarrhea (23.9%) were the most frequent adverse events, followed by other gastrointestinal symptoms such as nausea and vomiting (19.3%) and constipation (15.9%). There was a greater prevalence of eczema and itching experienced in boys (19.6%) than in girls (4.8%).

### 3.8. Behaviors after Experiencing Adverse Events

The behaviors of mothers after their children had experienced adverse events are shown in Table 8. Less than half of the mothers (40.9%) stopped supplementing their children immediately. Moreover, 18.2% of them reported that they did not act differently after their children experienced adverse events. Some of them reported the adverse events to relevant public institutions or authorities (14.8% to the National Consumer Affairs Center of Japan or other consumer affairs centers, 6.8% to the Ministry of Health, Labor and Welfare or Consumer Affairs Agency, Government of Japan, and 4.5% to public health centers), and more reports were made by the mothers of boys. In addition, 2.3% of mothers took their children to a hospital.

### 3.9. The Prevalence of Dietary Supplement Use by Mothers

We also asked mothers who gave dietary supplements to their children (*n* = 2439) whether they currently used or had previously used dietary supplements. Of these, 1594 mothers (65.4%) were currently using dietary supplements, 580 mothers (23.8%) had previously used dietary supplements in the past, and 265 mothers (10.9%) had never used dietary supplements.

### 3.10. The Perceptions Concerning Dietary Supplements Among Mothers

We assessed mothers’ perceptions of dietary supplements using a 14-topic questionnaire. The results are shown in Table 9. Although the most preferable answer was set as “Strongly disagree”, only from 5.4% to 38.2% of mothers answered correctly (disagree or strongly disagree) depending on the question items. Nearly 60% of mothers believed (strongly agree or agree) that “Dietary supplements made from natural ingredients or herbs are safe. (58.3%)”, “Dietary supplements made from foods are safe. (57.1%)”, and “Food additives should be avoided. (60.0%)”. Just over half of the mothers (54.8%) thought word of mouth from other users was more trustworthy (“I want to use highly recommended dietary supplement by users.”). In addition, 52.2% of mothers hoped that their children would learn about dietary supplements at school.

## 4. Discussion

In the present study, a nationwide survey of dietary supplement use in elementary, junior high, and high school students was conducted. The prevalence of current and previous dietary supplement use in overall school-aged children across Japan was 21.6% (current use, 16.4%; previous use, 5.2%), which was lower than for those of the same age in the United States (31–46.2%) [16,17], Italy (34.8%) [18], and Korea (34%) [19], but consistent with the results from Australia (20.1–23.5%) [20] and an urban Japanese city of Nara prefecture study (20.4%) [21]. Not only was the prevalence of dietary supplement use in each grade similar, but the increase in use in line with increasing age as shown in the Nara study was also replicated. In addition, Sato et al. reported the prevalence of dietary supplement use in preschool children as 8.0% [13], whereas the prevalence in college students and adults has been estimated to be between 32.0% and 60.7%, respectively [15,22]. Thus, our result of an increase from 15.3% in ES (1st-3rd) to 29.2% in HS falls in the middle of the reported range between preschoolers and college students. The trend in dietary supplement use has a positive correlation in the age.

The major purposes for dietary supplement use were shown to be maintenance of health and supplementation of nutrients. Since school-age children experience dynamic changes in body composition and height, insufficient nutritional intake may be a concern for mothers, as we have observed the popularity of growth-promoting supplements and cod liver oil especially in younger ages. Nearly half the mothers believed that supplementation can work well for children who are picky eaters, which is in line with a study that showed an unfavorable and unbalanced diet in children may be accompanied with dietary supplement use [13]. The previous reports concerning adolescents have shown males prefer protein/amino acids or performance related products [23], whereas females prefer body weight loss-related products [24,25]. The tendency of the purpose of dietary supplement use in boys is consistent with the aforementioned result; however, the use of weight loss products involved only 2.3% of the girls, even in the HS girls. This was likely due to this survey being conducted on mothers, who do not normally give their children dietary supplements for body weight loss, as seen in the popularity of growth-promoting supplements in this report. Having different purposes for dietary supplement use between boys and girls might have resulted in a higher prevalence in boys than in girls, as opposed to the higher prevalence use in women than men in adult age groups [15,22].

The prevalence of dietary supplement use in mothers who gave their children dietary supplements in this survey was 89.1% (65.4% currently using; 23.8% previous use), and this prevalence rate is greater than the prevalence rate for women in general. Sato et al. reported that the prevalence of dietary supplement use was higher among mothers who gave dietary supplements to their preschool children [13]. This indicates that dietary supplement use in mothers is associated with dietary supplement use in their children. Furthermore, given daughters are considered to be more affected by their mother’s behavior and attitudes than sons [26], it is possible that dietary supplement use in mothers may lead to chronic dietary supplement use in their daughters that, in turn, may lead to a higher prevalence of use in women.

A mother’s understanding of dietary supplements is important in relation to dietary supplement use in their child or children. Since mothers who use dietary supplements have been reported as being more educated with a strong awareness of eating needs [27], it would appear they are willing to learn about children’s nutrition to select the options they believe to be best-suited to their children. However, their knowledge of dietary supplements is mostly derived from the internet. In this survey, 40.4% of mothers obtained the information of dietary supplements through the internet. Previously, we also surveyed the information source in other subjects in Japan [15,28]. The internet was utilized by 38.3% of college students and 67.5% of general supplement users in adult ages. In addition, 19.6% of college students and 48.7% of general supplement users have purchased dietary supplements via the internet. In this regard, the internet plays an important role in dietary supplements. However, information on the internet is often not based on reliable scientific evidence and may lead to over-estimating the safety of dietary supplements [29,30]. In fact, inappropriate use by mothers such as concomitant use of multiple products and use of products intended for adults were observed in our survey. On the other hand, only 3.0% of mothers gained information from healthcare professionals (physicians, pharmacists, dietitians). There are some reports that show knowledge about dietary supplements in healthcare professionals was not enough [31,32], however, they believe know how to take care of children’s health via daily diet or with medicines instead of easily depending on dietary supplements. Appropriate information about the safety and the effectiveness of dietary supplements should be provided to them by not only health professionals via schools, sports clubs or other community associations, but also government websites, such as the Ministry of Health, Labor and Welfare or the Consumer Affairs Agency, Government of Japan.

The present survey also revealed that 3.6% of children had experienced adverse events, with a higher prevalence in younger school children. Previously, we reported that even physicians and pharmacists were unable to define cause-and-effect relationships for adverse events with dietary supplement use [22], so we could not evaluate cause-and-effect relationships for adverse effects in this survey. However, children have immature bodies and are in a state of physical development; thus, they are particularly vulnerable to adverse events, which have been reported to have occurred in children [14]. Since the products used by boys often contain proteins in high concentrations, mothers need to be aware of the risks of allergies before using such products. Moreover, cod liver oil products were shown to be popular in elementary school children in this survey. After World War II, malnutrition was a major problem, especially among children in Japan, and cod liver oil was recommended as rich in vitamin A and vitamin D. However, the situation now in terms of children’s nutritional intake has remarkably improved. At the present day, there are only few case reports about the health problems caused by vitamin A deficiency. These cases happened in specific situations, such as extreme picky eating, diarrhea, or virus infection. Meanwhile, there are few reports of the health problems caused by vitamin D deficiency. It is 1.1 per 100,000 among under people 15 years of age and 3.5 per 100,000 among people under 5 years of age [33]. On the other hand, a daily intake and high amounts of vitamin A from liver or vitamin supplements may lead to unwelcome side effects [34,35,36]. Furthermore, relatively high concentrations of dioxins in some fish oil supplements have been reported [37]. Dietary supplements contain various ingredients and are typically in a concentrated form. The efficacy and safety of these products, in addition to the risks involved when a child uses these products, remain unknown. In addition, the concomitant use of multiple products might contribute to increasing the risks of adverse events due to unknown interactions or excessive micronutrient intakes, especially when products are modified or adulterated through the addition of unmeasured elements, including iron [38]. It is important for mothers to report adverse events as soon as possible, but most mothers do not inform the appropriate authorities. Mothers need to be aware of the possibility of adverse events as a result of their children using dietary supplements, and what should be done in the event of such an adverse event.

The present study has several limitations. First, this study was conducted using an internet survey; thus, the participants of this study were not fully representative of the population of Japan. However, the mothers participating in this study were recruited from various regions of Japan. To the best of our knowledge, this study is the first nationwide survey focused on dietary supplement use among elementary, junior high, and high school children. Second, this was a cross-sectional study and we did not assess the diet quality of the participants’ children. Thus, we could not evaluate how dietary supplement use during their time at school might affect their subsequent dietary supplement use, dietary habits, or health in general.

## 5. Conclusions

We conducted a nationwide survey and revealed the prevalence of dietary supplement use among elementary school, junior high school, and high school students. Overall, 16.4% of children were currently using dietary supplements. The results of this study suggested that dietary supplement use among school-age children might be influenced by their mothers’ perceptions of dietary supplements. However, mothers tend to overestimate the safety of dietary supplements. Therefore, to reduce the risk of inappropriate use of dietary supplements and to prevent adverse events, further targeted education on dietary supplements for parents is essential.

## Figures and Tables

**Table 1:** Characteristics of the children of surveyed mothers.

	Preliminary Survey		Targeted Survey	
	*n*	%	*n*	%
All	19,041		2439	
Sex				
Boys	9791	51.4	1236	50.7
Girls	9250	48.6	1203	49.3
Grade ^1^				
ES (1st-3rd)	5264	27.6	590	24.2
ES (4th-6th)	4479	23.5	613	25.1
JHS	4291	22.5	618	25.3
HS	5007	26.3	618	25.3
Residential area				
Hokkaido	1078	5.7	145	5.9
Tohoku	1238	6.5	158	6.5
Kanto	6132	32.2	785	32.2
Chubu	3493	18.3	420	17.2
Kinki	3567	18.7	461	18.9
Chugoku	1137	6.0	145	5.9
Shikoku	489	2.6	63	2.6
Kyusyu	1907	10.0	262	10.7
Household income ^2^				
<2 million yen	773	4.1	90	3.7
2–4 million yen	3212	16.9	348	14.3
4–6 million yen	4886	25.7	625	25.6
6–8 million yen	3167	16.6	420	17.2
8–10 million yen	1434	7.5	252	10.3
10–12 million yen	633	3.3	98	4.0
12–15 million yen	289	1.5	49	2.0
15–20 million yen	98	0.5	22	0.9
>20 million yen	41	0.2	4	0.2
No answer	4508	23.7	531	21.8

^1^ ES: Elementary School, JHS: Junior High School, HS: High School; ^2^ An assumed exchange rate of 110 yen to one US dollar.

**Table 2:** The prevalence of dietary supplement use in children in the preliminary survey.

	Currently Using			Previously Used			Never Used			*p*-Value
	All 3127	Boys 1791	Girls 1336	All 998	Boy 553	Girls 445	All 14,916	Boys 7447	Girls 7469	
All (%)	16.4	18.3	14.4	5.2	5.6	4.8	78.3	76.1	80.7	<0.01
Grade ^1^ (%)										
ES (1st-3rd)	12.4	13.5	11.1	3.0	3.2	2.8	84.6	83.2	86.1	0.02
ES (4th-6th)	14.6	16.2	13.0	4.1	4.0	4.2	81.3	79.8	82.8	<0.01
JHS	17.5	20.2	14.9	6.0	6.6	5.5	76.5	73.3	79.7	<0.01
HS	21.3	23.8	18.7	7.9	9.0	6.8	70.8	67.2	74.4	<0.01

^1^ ES: Elementary School, JHS: Junior High School, HS: High School; All (*n* = 19,041), boys (*n* = 9791), girls (*n* = 9250); ES 1st-3rd grade (*n* = 5264, boys (*n* =2803), girls (*n* = 2461)); ES 4th-6th grade (*n* = 4479, boys (*n* = 2280), girls (*n* = 2199)); JHS (*n* = 4291, boys (*n* = 2151), girls (*n* = 2140)); HS (*n* = 5007, boys (*n* = 2557), girls (*n* = 2450)); Note: A chi-squared test was used to conduct statistical analyses between boys and girls.

**Table 3:** How do you get information about dietary supplements?

	All (*N* = 2439)	Boys (*N* = 1236)	Girls (*N* = 1203)	*p*-Value
Internet (%)	40.4	42.1	38.7	0.09
Stores (%)	19.7	19.2	20.3	0.49
Television (%)	18.1	17.4	18.9	0.35
Friends or acquaintances (%)	15.9	17.5	14.2	0.03
Product labels (%)	13.2	13.3	13.0	0.78
Family (%)	12.1	11.7	12.6	0.50
Pharmacists or drug store clerks (%)	10.1	8.7	11.5	0.03
Newspapers, magazines, flyers (%)	6.5	6.6	6.3	0.75
Clinic (physicians, pharmacists, dietitians) (%)	3.0	2.4	3.5	0.12
Inquiry to the manufacturer (%)	2.5	2.8	2.2	0.42
Coaches of clubs (%)	2.3	3.7	0.7	<0.01
School teachers (%)	1.7	2.1	1.3	0.14
Radio (%)	1.2	1.2	1.2	0.91
Others (%)	4.6	4.7	4.5	0.81

Note: Multiple answers; Statistical analyses were conducted to compare the differences between boys and girls using a chi-squared test.

**Table 4:** How do you obtain dietary supplements?

	All (*N* = 2439)	Boys (*N* = 1236)	Girls (*N* = 1203)	*p*-Value
Pharmacy or drugstore (%)	42.4	39.0	46.0	<0.01
Internet (%)	40.6	42.3	38.8	0.08
Mail order (except internet shopping) (%)	11.2	12.1	10.2	0.13
Supermarket (%)	7.9	8.3	7.5	0.48
Convenience store (%)	2.3	2.3	2.2	0.87
Friends or acquaintances (%)	2.3	1.9	2.7	0.15
Sports store (%)	2.1	3.2	0.8	<0.01
Co-op ^1^ store (%)	1.7	1.6	1.7	0.81
School (%)	1.4	1.5	1.2	0.65
Department store (%)	1.3	0.9	1.7	0.06
Sports club (%)	0.6	0.9	0.2	0.04
Others (%)	4.9	4.9	4.8	0.90

^1^ Co-op: consumers’ cooperative; Note: Multiple answers; Statistical analyses were conducted to compare the differences between boys and girls using a chi-squared test.

**Table 5:** What is the purpose of dietary supplement use?

	Boys (*n* = 1236)						Girls (*n* = 1203)					
	*N*	ES 1st-3rd (309) (%)	ES 4th-6th (309) (%)	JHS (309) (%)	HS (309) (%)	*p*-Value	*N*	ES 1st-3rd (281) (%)	ES 4th-6th (304) (%)	JHS (309) (%)	HS (309) (%)	*p*-Value
Maintenance of health	621	61.2	49.8	46.6	43.4	<0.01	677	61.2	55.6	49.2	59.5	0.01
Supplementation of nutrients	528	47.2	42.7	43.7	37.2	0.09	582	48.4	45.4	47.9	51.8	0.47
Enhance growth	475	31.4	45.3	48.9	28.2	<0.01	271	26.3	30.9	25.2	8.1	<0.01
Improvements to health	198	14.6	17.8	14.2	17.5	0.49	208	13.5	15.1	17.5	22.7	0.02
Enhance stamina	259	12.0	15.5	23.6	32.7	<0.01	134	8.5	10.9	12.6	12.3	0.38
Prevention of diseases	151	14.2	10.4	12.0	12.3	0.53	184	16.4	17.4	14.6	12.9	0.43
Enhance athletic performance	144	2.6	8.4	15.9	19.7	<0.01	51	0.7	3.6	7.1	5.2	<0.01
Improve academic performance	69	8.7	3.9	5.5	4.2	0.03	65	5.3	6.6	4.2	5.5	0.64
Treatment of diseases	31	1.9	2.9	2.9	2.3	0.83	34	3.9	3.6	2.3	1.6	0.27
Weight loss	27	2.9	1.6	2.6	1.6	0.59	34	1.8	2.0	2.9	4.5	0.16
Others	22	2.9	1.3	1.3	1.6	0.37	28	2.1	2.0	1.6	3.6	0.40

ES: Elementary School, JHS: Junior High School, HS: High School; Note: Multiple answers. Statistical analyses were conducted among groups using a chi-square test.

**Table 6:** What kind of dietary supplements are you giving your child?

	Boys (*n* = 1236)						Girls (*n* = 1203)					
	*n*	ES 1st-3rd (309) (%)	ES 4th-6th (309) (%)	JHS (309) (%)	HS (309) (%)	*p*	*n*	ES 1st-3rd (281) (%)	ES 4th-6th (304) (%)	JHS (309) (%)	HS (309) (%)	*p*-value
**Vitamin/Mineral**												
Multi-vitamins and minerals	28	2.3	1.9	3.6	1.3	0.28	36	3.6	2.0	3.2	3.2	0.68
Multi-vitamins	97	7.1	6.1	10.0	8.1	0.32	116	7.5	8.9	7.4	14.6	0.01
Individual vitamin	122	10.7	9.1	7.8	12.0	0.32	174	12.1	14.1	12.6	18.8	0.08
Multi-minerals	7	0.0	1.3	0.6	0.3	-	31	2.1	2.3	1.6	4.2	0.19
Individual mineral	229	17.8	16.8	22.3	17.2	0.26	240	14.9	14.5	23.3	26.5	<0.01
Any type	409	32.4	31.1	37.5	31.4	0.28	505	36.3	35.2	42.1	53.7	<0.01
**Non-Vitamin, Non-Mineral**												
Growth-promoting	214	15.9	23.9	20.4	9.1	<0.01	115	12.1	13.8	11.3	1.3	<0.01
Protein/ Amino acid	235	7.1	14.9	20.7	33.3	<0.01	71	4.6	5.6	8.4	4.9	0.17
Probiotics	91	7.8	10.0	6.5	5.2	0.12	90	10.7	8.6	6.1	4.9	0.04
*n*-3 PUFA	76	5.5	5.8	6.5	6.8	0.91	52	4.3	4.3	3.9	4.9	0.95
Cod liver oil	60	12.9	3.9	1.3	1.3	<0.01	54	12.8	3.3	1.6	1.0	<0.01
Aojiru ^1^	42	5.5	3.9	2.3	1.9	0.06	51	6.0	4.6	3.6	2.9	0.26
Blueberry/Lutein	46	1.9	4.2	4.5	4.2	0.30	49	2.1	3.6	6.8	3.6	0.03
Weight loss	2	0.0	0.0	0.3	0.3	-	9	0.4	0.0	0.3	2.3	-
Others	306	24.9	22.7	22.7	28.8	0.24	407	23.8	32.9	36.2	41.4	<0.01

ES: Elementary School, JHS: Junior High School, HS: High School, PUFA: polyunsaturated fatty acid; ^1^ A powdered drink mix made from green leafy vegetables such as young leaves of *Angelica keiskei* (Miq.) Koidz and Barley, and *Brassica oleracea* L. var. *acephala* DC; Note: Multiple answers. Statistical analyses were conducted among groups using a chi-square test.

**Table 7:** Has your child ever experienced adverse events due to dietary supplement use? If yes, what symptom(s) did your child experience?

	All (2439) (%)	Boys (1236) (%)	Girls (1203) (%)	*p*-Value	ES 1st-3rd (590) (%)	ES 4th-6th (613) (%)	JHS (618) (%)	HS (618) (%)	*p*-Value
Never	96.4	96.3	96.5		94.6	96.6	96.4	97.9	
Yes	3.6	3.7	3.5	0.76	5.4	3.4	3.6	2.1	0.02
Symptom ^1^									
Stomachache	23.9	21.7	26.2	0.63	21.9	19.0	13.6	23.1	0.45
Diarrhea	23.9	26.1	21.4	0.61	6.3	4.8	4.5	15.4	0.81
Nausea & Vomiting	19.3	15.2	23.8	0.31	31.3	14.3	18.2	30.8	-
Constipation	15.9	15.2	16.7	0.85	18.8	28.6	27.3	23.1	-
Eczema & Itching	12.5	19.6	4.8	0.04	15.6	19.0	18.2	7.7	-
Headache	6.8	4.3	9.5	-	12.5	4.8	18.2	15.4	-
Fatigue	6.8	6.5	7.1	-	9.4	9.5	4.5	0	-
Palpitations	2.3	4.3	0.0	-	0	4.8	4.5	0	-
Others	4.5	6.5	2.4	-	0	9.5	9.1	0	-

ES: Elementary School, JHS: Junior High School, HS: High School; ^1^ All (*n* = 88), Boys (*n* = 46), Girls (*n* = 42), ES 1st-3rd grade (*n* = 32), ES 4th-6th grade (*n* = 21), JHS (*n* = 22), HS (*n* = 13); Note: Multiple answers; Statistical analyses were conducted between the groups.

**Table 8:** How did you respond to the adverse events?

	All (88)	Boys (46)	Girls (42)	ES 1st-3rd (32)	ES 4th-6th (21)	JHS (22)	HS (13)
Did not act differently (%)	18.2	17.4	19.0	9.4	23.8	22.7	23.1
Stopped using dietary supplements immediately (%)	40.9	37.0	45.2	34.4	42.9	54.5	30.8
Reported the incident to the National Consumer Affairs Center of Japan or another consumer center (%)	14.8	21.7	7.1	18.8	9.5	9.1	23.1
Complained to the manufacturer (%)	13.6	15.2	11.9	25.0	9.5	4.5	7.7
Complained to the retail store (%)	13.6	13.0	14.3	18.8	14.3	13.6	0.0
Reported the incident to the Ministry of Health, Labor and Welfare or Consumer Affairs Agency, Government of Japan (%)	6.8	8.7	4.8	9.4	0.0	4.5	15.4
Reported the incident to a public health center (%)	4.5	4.3	4.8	9.4	4.8	0.0	0.0
Went to a hospital (%)	2.3	2.2	2.4	3.1	4.8	0.0	0.0
Others (%)	3.4	4.3	2.4	0.0	0.0	4.5	15.4

ES: Elementary School, JHS: Junior High School, HS: High School; Note: Multiple answers.

**Table 9:** Perceptions of dietary supplements among mothers.

	Strongly Agree	Agree	Neither Agree nor Disagree	Disagree	Strongly Disagree
Dietary supplements are safe because they are just foods. (%)	5.6	44.0	41.1	7.5	1.8
Dietary supplements made from natural ingredients or herbs are safe. (%)	7.5	50.8	34.7	5.7	1.4
Food additives should be avoided. (%)	14.9	45.1	33.5	5.1	1.3
Dietary supplements made from foods are safe. (%)	7.0	50.1	37.6	4.2	1.2
The effectiveness of commercial dietary supplements are confirmed and reliable. (%)	3.8	30.8	54.3	9.2	1.9
I want to use highly recommended dietary supplement by users. (%)	8.4	46.4	35.4	7.5	2.3
Products recommended by health professionals are effective. (%)	3.5	24.7	49.0	16.6	6.1
Dietary supplements can be used concomitantly with drugs. (%)	3.8	22.9	45.2	20.1	8.0
Dietary supplements can help prevent diseases. (%)	4.3	34.6	42.6	13.9	4.5
Dietary supplements can treat diseases. (%)	2.8	17.4	41.5	25.0	13.2
Children who are picky eaters should take dietary supplements to supplement nutrition. (%)	6.2	38.7	37.5	12.7	4.9
Pregnant women should take dietary supplements to supplement nutrition. (%)	7.3	38.4	39.4	11.1	3.9
I want to use weight loss or muscle building dietary supplements. (%)	8.5	33.2	34.9	14.5	9.0
Children should learn about dietary supplements at school. (%)	9.0	43.2	36.0	8.9	3.0
